# Supplementary material for: E-CLEAP: An ensemble learning model for efficient and accurate identification of antimicrobial peptides
Source: PLoS One. 2024 May 9;19(5):e0300125. doi: 10.1371/journal.pone.0300125 (PMC11081394; doi:10.1371/journal.pone.0300125)
Supplement: Attachment — Submitted filename: Respond reviewer.docx [file pone.0300125.s001.docx]

Response to Reviewer 1 Comments

The revised paragraphs based on REVIEWER #1 is labeled in yellow.

| **No.** | **Reviewer comment** | **Author response** | **Change made** |
| --- | --- | --- | --- |
| 1 | The literature review of the paper seems really poor, the authors are suggested to add the the latest computational models developed for antimicrobial peptides papers such as, LMPred, AMPlify, ClassAMP, AI4AMP, AmPEP, AMP-GSM, and many more in order to strengthen the literature review section. | Thanks for your comment. I consider this to be a valuable suggestion. Following the reviewer's recommendation, I have included the AI4AMP, AmPEP, AMP-GMS, and AMPlify models to enhance the literature review. | Page 3, Line 41-49  Page 16-17, Line 340-349 |
| 2 | As the author used ensemble learning model to increase the prediction of the model, however in the introduction section the authors didn't mentioned the effectiveness and motivation to use ensemble learning as provided in iAFPs-EnC-GA and iAtbP-Hyb-EnC models. | Thank you for your suggestion. I have revised the relevant sentence in line 54 and added references to support this point. I have elaborated on the motivation and effectiveness of using ensemble learning models. | Page 3, Line 54-58  Page 17, Line 350-355 |
| 3 | The authors are suggested to provide a paragraph related to smote oversampling with its proper algorithm. | Thank you for your suggestion. I think this is an excellent suggestion. Following the reviewer's advice, we have incorporated additional content regarding SMOTE oversampling and its appropriate algorithms. | Page 10, Line 179-185 |
| 4 | The should provide the results of the model before and after applying smote. | Thank you for your comment. As you have expressed concerns, the results can be presented more clearly. In accordance with your suggestion, I have added a comparison of the results of the E-CLEAP model before and after using the SMOTE algorithm, along with the inclusion of Figure 3 and Table 1. | Page 10, Line 186-192 |
| 5 | The authors should provided the size of the training vector before training a machine learning model. | Thank you for your question. The author recognized this deficiency and, following your suggestion, provided the size of the training vector before training the machine learning model. | Page 10, Line 194-195  Page 12, Line 220-221 |
| 6 | The results of the proposed model should be compared with existing models in terms of predictive results and time complexity. | Thank you very much for your professional review of the article. In the manuscript, I have provided a detailed comparison between the performance metrics of the E-CLEAP model, including ROC curves, AUC, ACC, and those of traditional SVM, DT, KNN, and Bayes classifiers. In the Discussion section, I have added a new paragraph to clearly articulate the superior performance highlighted by comparing the E-CLEAP model with traditional models. | Page 15, Line 278-282 |

Response to Reviewer 2 Comments

The revised paragraphs based on REVIEWER #1 is labeled in yellow.

| **No.** | **Reviewer comment** | **Author response** | **Change made** |
| --- | --- | --- | --- |
| 1 | In the introduction section the author are advised to clearly mention the novelty of the proposed model. | Thank you for your valuable suggestion. I have incorporated the novelty of the proposed model into the introduction section. | Page 4, Line 64-66 |
| 2 | To measure the generalization power and overfitting of the proposed model, an independent dataset is highly mandatory. | I highly appreciate your perspective and thank you for your professional review. Regarding the dataset I utilized, I would like to provide the following clarification: the test set is an independent dataset, and during the training process, I further split the training set into training and validation sets for five-fold cross-validation. |  |
| 3 | the ShAp and LIME analysis of the proposed model is required to evaluate the contribution of the extracted features. | Thank you for your suggestion. I consider this to be a valuable suggestion. Following the reviewer's advice, I have incorporated LIME analysis to assess the contribution of features extracted by the E-CLEAP model. Regarding the choice of LIME analysis, I provide the following explanation: Multilayer Perceptrons (MLP) are non-tree models, and the support for the 'shap' library in Python is relatively weaker for non-tree models. Therefore, the 'lime' library is chosen for interpreting non-tree models. | Page 13-14, Line 247-263 |
| 4 | An extra section are needs to incorporated related to majority voting based ensemble learning. | Thank you for your suggestion. As suggested by the reviewer, I have added a section in the methodology that provides a detailed explanation of why the soft voting mechanism was chosen and elaborates on the ensemble learning model with a majority voting mechanism. There are two common voting mechanisms, and I believe it is necessary to explain the rationale behind the choice. This adds a level of rigor to the methodology. | Page 6, Line 112-121 |
| 5 | A pseudo code of the proposed model should be added. | Thank you very much for your suggestion. As you are concerned, pseudocode can make the model more clearly presented. I have added the pseudocode of my proposed model in the methodology section. | Page 6-7, Line 121-122 |
| 6 | A github link should be provided in the manuscript regarding the used datasets and the codes of the proposed model. | I greatly appreciate your professional review of my article. Additionally, I have included a GitHub link in the abstract section, providing access to the dataset used and the code for the proposed model. | Page 2, Line 23 |
